# Supplementary material for: Assessment of Dietary Sodium, Potassium and Sodium-Potassium Ratio Intake by 72 h Dietary Recall and Comparison with a 24 h Urinary Sodium and Potassium Excretion in Dominican Adults
Source: Nutrients. 2025 Jan 24;17(3):434. doi: 10.3390/nu17030434 (PMC11820599; doi:10.3390/nu17030434)
Supplement: Supplementary file 1 [file nutrients-17-00434-s001.zip › nutrients-3441747-supplementary.pdf]

Table S1. Foods whose composition was taken from the nutritional information provided by the manufacturer, supermarket or app.

Agave [6*]	Cracker – soda s/salt [*]
Apple compote ("Compota" Be Plus®) [*]	Cream – sour [*]
Bananas - Cariba® - very thinly fried green plantains or tostones [***]	Cutlet - smoked [*]
Beverage - malt beverage [*]	Dominican salami [*]
Biscuits – butter [***]	Flour ("el Negrito"®) / semolina [*]
Biscuit – Oreo [*]	Flour tortillas for tacos [****]
Bread - flaxseed [6*]	Focaccia sticks [*]
Bread - pita [****]	Juice – chinola [*]
Bread - Mini pittetes toufuyan® [***]	Juice – pear [6*]
Bread - sobao [*]	Longaniza sausage [*]
Bread - sourdough (dough) [****]	Milk - almond and rice [*]
Brownies [6*]	Milk – nuts [5*]
Butter - almond [*]	Pancake - oatmeal [6*]
Cake - sultana sponge [*]	Peas - green pigeon [*]
Calzone - ham and cheese [6*]	Pigeon peas ("guandules") with coconut/canned [*]
Candies - ginger mint [***]	Pork and mango? dumplings ("mangfou") [****]
Cassava – fried [****]	Potato supreme [****]
Cassava with garlic [*]	Rice (Manna pack®) [6*]
Cheese -cream [*]	Sausages - artisan pork [****]
Cheese – Danish [6*]	Sausages - Vienna [6*]
Cheese - frying [*]	Seasoning powder [*]
Cheese - Geo [*]	Seasoning - liquid [*]
Cheese – leaf [*]	Soft drink - Diet Coke [6*]
Cheese ravioli [****]	Soft drink - Gatorade [*]
Cheetos/cheese stick [*]	Soft drink - 7up [*]
Chiken baker roll [**]	Soybean - meat textured [*]
Chicken Meatball [6*]	Spinach dip /cakes [6*]
Chicken Ramen [6*]	Snickers®: milk chocolate and peanuts [6*]
Cereal – Nesquick® [*]	Seitan - (Natursoy-Seitan®. Seitán= wheat gluten-based food preparation) [5*]
Cereal - Vitalisimo® [*]	Tea - green [6*]
Chicharrón + lemon Frito lay [*]	"Tortilla" wrap Toufuyan® [*]
Chocolate ("barrita" to dilute) [*]	
Chocolate – white, caramel [****]	
Coffee creamer/cream [*]	
Crackers cheese - Ritz® [***]	

[*] manufacturer, [**] PriceSmart https://www.pricesmart.com/es-do

[***] MyFitnessPal (app) https://www.myfitnesspal.com/es

[****] Fat Secret Esp (app) https://www.fatsecret.es/

[5*] FAT – Food at time (app) https://fat-food-at-time.softonic.com/android

[6*] Open Food facts https://es.openfoodfacts.org/

Table S2. Food composition obtained from the INCAP [27] and USDA [28] Food Composition Tables included in the DIAL software.

Agave - nectar [28]<br>Bread - bagel [28]<br>Celery/yellow beet strain [27]<br>Cheese - cake [28]<br>Cheese –yellow, sliced [27]<br>Chicken burrito [27]<br>Chia- seeds [28]<br>Chicken - breaded boneless (KFC®) [27]<br>Chicken offal [27]<br>Chili w/meat [27]<br>Coffee - espresso [27]<br>Cow's foot/beef/feed [27]<br>Doughnuts w/ sugar [27]<br>Egg - boiled/ hard boiled [27]<br>Eggs - scrambled [27]<br>"Manzana de oro" (Spondias citerea) [27]<br>Maple syrup [27]	Milk cream [27]<br>Nacho with cheese and beans ("Cielito lindo") [27]<br>Orange - sour [27]<br>Pasta – sauce, processed [27]<br>Pear – nectar, canned [27]<br>Pizza -pepperoni [27]<br>Ranch sauce [27]<br>Ranch style tomato sauce/ Tostitos [27]<br>Sandwich spread [27]<br>Soursop ("guanábana")[27]<br>Sweetbreads - other/ cooked [27]<br>Sweetbreads - raw [27]<br>Taco bell burrito original w/ meat [27]<br>Tomato paste – canned s/salt [27]<br>Tostitos: nacho-flavoured corn tostadas [27]<br>Yam ("ñame") - cooked s/salt [27]<br>Yucca /root [27]
